# Embryogenesis of flattened colonies implies the innovation required for the evolution of spheroidal colonies in volvocine green algae

**DOI:** 10.1186/s12862-019-1452-x

**Published:** 2019-06-11

**Authors:** Shota Yamashita, Hisayoshi Nozaki

**Affiliations:** Department of Biological Sciences, Graduate School of Science, University of Tokyo, 7-3-1 Hongo, Bunkyo-ku, Tokyo, 113-0033 Japan

**Keywords:** Multicellularity, Body plan, Embryogenesis, Volvocine green algae, *Gonium*, *Tetrabaena*

## Abstract

**Background:**

Volvocine algae provide a suitable model for investigation of the evolution of multicellular organisms. Within this group, evolution of the body plan from flattened to spheroidal colonies is thought to have occurred independently in two different lineages, Volvocaceae and *Astrephomene*. Volvocacean species undergo inversion to form a spheroidal cell layer following successive cell divisions during embryogenesis. During inversion, the daughter protoplasts change their shape and develop acute chloroplast ends (opposite to basal bodies). By contrast, *Astrephomene* does not undergo inversion; rather, its daughter protoplasts rotate during successive cell divisions to form a spheroidal colony. However, the evolutionary pathways of these cellular events involved in the two tactics for formation of spheroidal colony are unclear, since the embryogenesis of extant volvocine genera with ancestral flattened colonies, such as *Gonium* and *Tetrabaena*, has not previously been investigated in detail.

**Results:**

We conducted time-lapse imaging by light microscopy and indirect immunofluorescence microscopy with staining of basal bodies, nuclei, and microtubules to observe embryogenesis in *G. pectorale* and *T. socialis*, which form 16-celled or 4-celled flattened colonies, respectively. In *G. pectorale*, a cup-shaped cell layer of the 16-celled embryo underwent gradual expansion after successive cell divisions, with the apical ends (position of basal bodies) of the square embryo’s peripheral protoplasts separated from each other. In *T. socialis*, on the other hand, there was no apparent expansion of the daughter protoplasts in 4-celled embryos after successive cell divisions, however the two pairs of diagonally opposed daughter protoplasts shifted slightly and flattened after hatching. Neither of these two species exhibited rotation of daughter protoplasts during successive cell divisions as in *Astrephomene* or the formation of acute chloroplast ends of daughter protoplasts as in volvocacean inversion.

**Conclusions:**

The present results indicate that the ancestor of *Astrephomene* might have newly acquired the rotation of daughter protoplasts after it diverged from the ancestor of *Gonium*, while the ancestor of Volvocaceae might have newly acquired the formation of acute chloroplast ends to complete inversion after divergence from the ancestor of Goniaceae (*Gonium* and *Astrephomene*).

**Electronic supplementary material:**

The online version of this article (10.1186/s12862-019-1452-x) contains supplementary material, which is available to authorized users.

## Background

The evolution of multicellular organisms from unicellular ancestors is one of the most important events in the history of life, known as evolutionary transitions in individuality [1]. Such transitions to multicellularity have occurred independently at least 25 times, in many different eukaryotic and prokaryotic branches [2]. In many lineages of multicellular organisms, including animals and land plants, the emergence of multicellularity was accompanied by the invention of embryogenesis, a developmental program for building a complicated body plan from a single cell [3]. An investigation into the evolution of embryogenesis would provide some clues to understand the complex and divergent body plans found in multicellular organisms. However, many multicellular lineages lack extant intermediates between unicellular and multicellular organisms with complex body plans, which makes it difficult to resolve the first steps in the evolution of embryogenesis.

**Fig. 1:**
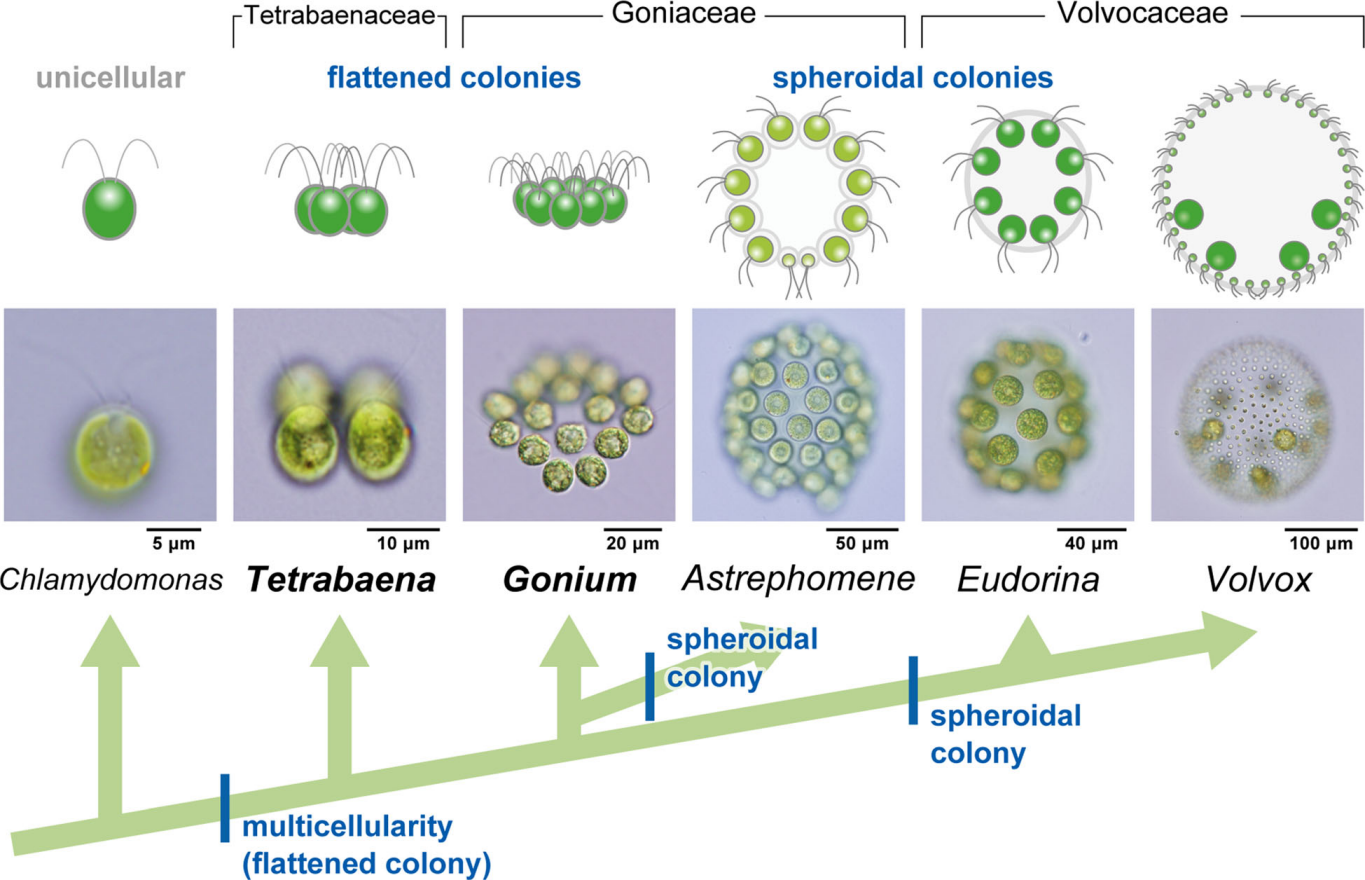
Schematic representation of the phylogenetic relationships of volvocine green algae and evolution of their body plans. Volvocine green algae consist of organisms of various complexities, ranging from unicellular *Chlamydomonas reinhardtii* to multicellular *Volvox*, which exhibits germ-soma differentiation. The evolution of spheroidal colonies is thought to have occurred twice, in the ancestors of *Astrephomene* and in those of Volvocaceae [4–6]. The formation of spheroidal colonies during embryogenesis is based on different cellular mechanisms in the two lineages (Additional file 6: Figure S1) [7]. There are two extant lineages with ancestral flattened colonies, the genus *Gonium* and the family Tetrabaenaceae. The phylogeny is based on a previous report [5]. All drawings and photographs represent lateral views of individuals with anterior sides (the direction of swimming) oriented toward the top of the figure. The photographs of *Tetrabaena*, *Astrephomene*, and *Volvox* are from a previous study [7]. The other photographs are original

This problem could be solved using a model lineage for the evolution of multicellularity, the volvocine green algae (Fig. 1) [8]. The volvocine green algae, which consist of Volvocaceae, Goniaceae, Tetrabaenaceae and their closest unicellular relatives [8], include organisms of various intermediate stages of organismal complexity, ranging from unicellular *Chlamydomonas* to multicellular *Volvox*, which exhibits germ-soma differentiation. The multicellular genera in this lineage have simple body plans consisting of a flattened or spheroidal monolayer of *Chlamydomonas*-like cells, and arose from unicellular ancestors approximately 200 million years ago [9]. This is a more recent event than the emergence of animals more than 600 million years ago [10, 11], or the emergence of land plants approximately 500 million years ago [12]. Additionally, as the volvocine green algae *C. reinhardtii* and *V*. *carteri* are often used as model organisms, genome sequence data [13, 14] and diverse tools—including those related to cultivation, molecular biology, and genetics—are readily available. The genome sequence data of other genera in the volvocine lineage, such as *Gonium pectorale* [15], *Tetrabaena socialis* [16], *Eudorina* sp., and *Yamagishiella unicocca* [17], have also been made available in recent years. Thus, this lineage would be a suitable model for use in research on the evolution of embryogenesis and its role in bringing about the evolution of a complex body plan from a simpler one. There are several other colonial or multicellular lineages in Volvocales, including *Stephanosphaera*, *Pascherina* and *Pyrobotrys*, but they are all phylogenetically separated from the volvocine lineage [18, 19].

The volvocine algae has undergone parallel evolution of body plans, with two independent transitions from a flattened to spheroidal colony, in Volvocaceae and the goniacean *Astrephomene* (Fig. 1) [4–6]. Though the two lineages are alike in the morphology of their spheroidal colonies, they differ in their tactics for forming these colonies during embryogenesis: the embryos of volvocacean species undergo inversion, while the embryos of *Astrephomene* undergo rotation of daughter protoplasts (Additional file 6: Figure S1) [7].

Volvocacean species form spheroidal colonies by inversion, a unique morphogenetic event that occurs during embryogenesis (Additional file 6: Figure S1a)*.* In volvocacean species, a mature reproductive cell initiates embryogenesis with successive cell divisions and forms a cup-shaped or hollow, spheroidal embryo composed of a single layer of daughter protoplasts laterally interconnected with one another by cytoplasmic bridges. Immediately following the successive divisions, the apical ends of daughter protoplasts (the side with basal bodies) are oriented toward the inside of the cell layer. The embryo then inverts its cell layer to form a spheroidal daughter colony with the apical ends of daughter protoplasts positioned on the outside. Developmental studies using *V*. *carteri* have revealed that the principal factors producing the force for folding the cell layer during inversion are the change in cell shape to flask-shaped with a stalk at the chloroplast ends (the opposite side from apical ends) of daughter protoplasts and the movement of protoplasts relative to cytoplasmic bridges (observed as relocation of cytoplasmic bridges to the stalks at chloroplast ends) [20–22]. The change in cell shape with the development of stalks or acute chloroplast ends and the location of cytoplasmic bridges at the chloroplast ends during inversion have also been reported in other *Volvox* species [23–26] and in other genera in Volvocaceae [27–31], which suggests that the volvocacean genera use common cellular mechanisms for inversion.

In contrast to Volvocaceae, *Astrephomene* does not employ inversion to construct a spheroidal daughter colony during embryogenesis (Additional file 6: Figure S1b) [7]. During successive cell divisions in the embryogenesis of *Astrephomene*, daughter protoplasts rotate outward, with the apical ends of protoplasts moving from the anterior (the side with flagella of the mother cell and the direction of swimming of the daughter colony) to posterior region at the outer (apical) surface of the cell layer. After the successive cell divisions, the cell layer forms a nearly spheroidal shape with the apical ends of the daughter protoplasts oriented toward the outside. Therefore, no inversion occurs in the embryogenesis of *Astrephomene.*

How these two different mechanisms for making spheroidal colonies evolved in Volvocaceae and *Astrephomene* is currently unclear, since the embryogenesis of extant genera with ancestral flattened colonies, such as *Gonium* and *Tetrabaena* (Fig. 1), has not been investigated in detail from this point of view. Though embryogenesis has been observed in *Gonium* and *Tetrabaena* by light microscopy time-lapse imaging [32–34] and transmission electron microscopy [33, 34], it is currently unclear whether the rotation of daughter protoplasts seen in *Astrephomene* or the cell shape change of Volvocaceae occurs in these genera, as thus far their embryogenesis has only been observed at certain stages and from limited angles.

In this study, we focused on the formation of flattened colonies in *G. pectorale* and *T. socialis* to evaluate whether they employ cellular mechanisms that may have been co-opted to make spheroidal colonies in Volvocaceae and *Astrephomene*. We conducted light microscopy time-lapse imaging showing all successive stages of embryogenesis, as well as indirect immunofluorescence microscopy staining basal bodies, nuclei, and microtubules of both species to investigate their embryogenesis at cellular and subcellular levels. Our results define the similarities and differences in the developmental events that lead to the formation of flattened and spheroidal colonies, thus providing clues to the evolutionary pathway from flattened to spheroidal colonies.

## Results

### Time-lapse imaging of embryogenesis in *G. pectorale* and *T. socialis*

Successive images of embryogenesis in *G. pectorale* from the anterior view (Additional file 1) and the anterior-lateral view (Additional file 2) were obtained and analyzed as movies. In *G. pectorale*, each cell in vegetative colonies performed four successive cell divisions to form 16-celled embryos (Additional file 6: Figure S2a–e). The cleavage patterns were as described in previous studies on *G. pectorale* [35–37]: the third cell division occurred nearly parallel to the first division to form an 8-celled embryo with a pattern of four zigzag rows of two protoplasts each (Additional file 6: Figure S2d), then the fourth cell division occurred nearly parallel to the second division to form a 16-celled embryo with four central protoplasts (A_3_, A_3_’, B_3_, and B_3_’ in Additional file 6: Figure S2e) and 12 peripheral protoplasts (Additional file 6: Figure S2e).


**Additional file 1:** **Movie S1.** Light microscopy time-lapse imaging of embryogenesis in *G. pectorale* from the anterior view. Scale bar: 5 μm, 900x speed. (AVI 2549 kb)



**Additional file 2: Movie S2.** Light microscopy time-lapse imaging of embryogenesis in *G. pectorale* from the anterior-lateral view. Scale bar: 5 μm, 900x speed. (AVI 2475 kb)


**Fig. 2:**
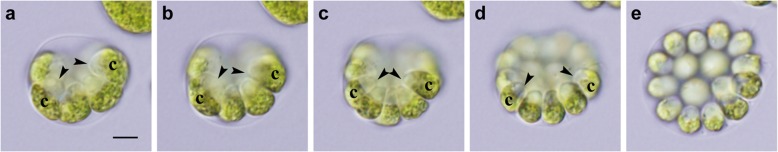
Successive cell divisions and expansion of the cell layer in embryogenesis of *G. pectorale*. Successive stages of an embryo observed by time-lapse analysis from the anterior-lateral view (Additional file 2). All images are at the same magnification. Scale bars: 5 μm. Note the longitudinal axis of each daughter protoplast, indicated by the positions of apical ends (*arrowheads*) and chloroplasts (*letter c*). **a** Early 8-celled stage. **b** Late 8-celled stage. **c** Early 16-celled stage. Rotation of daughter protoplasts is not observed during cell divisions (**a**–**c**). **d** Mid 16-celled stage during partial inversion. The cup-shaped cell layer of the embryo has expanded and the apical ends of outer daughter protoplasts (*arrowheads*) have separated from one another and moved outwards. **e** Late 16-celled stage, just prior to hatching. The cell layer has increasingly expanded and rotated slightly to show an almost anterior view


A time-lapse analysis of the anterior-lateral view of embryogenesis in *G. pectorale* (Fig. 2, Additional file 2) showed that the outward rotation of daughter protoplasts, as observed in *A*. *gubernaculifera* [7], did not occur during successive cell divisions, and the apical ends of the daughter protoplasts (transparent region opposite to the green chloroplasts) did not separate from one another (Fig. 2a, b). As a result, a cup-shaped cell layer with the apical ends of daughter protoplasts positioned inside the concave surface of the cup-shaped 16-celled embryo was formed just after the fourth cell division (Fig. 2c). At this point, each daughter protoplast appeared to be pear-shaped, with an acute apical end and a distended chloroplast end. The concave surface of the cup-shaped embryo then expanded, with the longitudinal axes of the 12 peripheral daughter protoplasts inclining toward the outside of the embryo (Fig. 2d). During this expansion, each daughter protoplast began to extend flagella from the apical end and changed its shape from pear-shaped to ovoid (Fig. 2d). The formation of stalks or acute chloroplast ends, seen during inversion in volvocacean species [7, 20, 23–31], was not observed in daughter protoplasts during this stage. Just prior to hatching, the cell layer of daughter colonies was still concave or distorted and did not fully expand within the mother cell wall (Fig. 2e). This distortion of the cell layer dissolved and the daughter colony fully expanded after it hatched out of the mother cell wall (Additional file 3), as described in previous reports [35, 38].

**Additional file 3: Movie S3.** Light microscopy video showing hatching of the daughter colony in *G. pectorale*. Scale bar: 10 μm, 1x speed. (MP4 8750 kb)


Successive images of embryogenesis in *T. socialis* from the anterior view (Additional file 4) and the anterior-lateral view (Additional file 5) were also obtained and analyzed as movies. The cleavage patterns in *T. socialis* (Additional file 6: Figure S2f–h) were identical to those previously observed [34]. In the 4-celled embryo just after successive cell divisions, one pair of diagonally opposed daughter protoplasts (B and B′ in Additional file 6: Figure S2 h) were slightly attached to each other at the center of the embryo, while the other pair were separated from each other (A and A’ in Additional file 6: Figure S2 h). A similar daughter protoplast arrangement was also observed in the 4-celled embryos of *G. pectorale* (Additional file 6: Figure S2c) and has been observed in other species of volvocine algae [32].


**Additional file 4: Movie S4.** Light microscopy time-lapse imaging of embryogenesis in *T. socialis* from the anterior view. Scale bar: 5 μm, 1,800x speed. (AVI 2090 kb)



**Additional file 5: Movie S5.** Light microscopy time-lapse imaging of embryogenesis in *T. socialis* from the anterior-lateral view. Scale bar: 5 μm, 1,800x speed. (AVI 1941 kb)


**Fig. 3:**
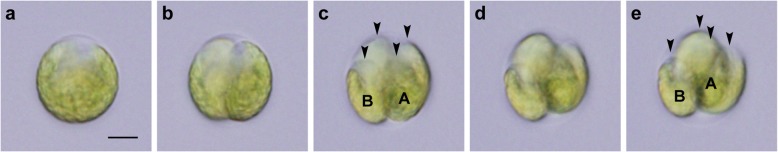
Successive cell divisions and slight shifting of daughter protoplasts during embryogenesis of *T. socialis*. Successive stages of an embryo observed by time-lapse analysis from the anterior-lateral view (Additional file 5). All images are at the same magnification. Scale bars: 5 μm. Note the longitudinal axis of each daughter protoplast, indicated by the positions of apical ends (*arrowheads*) and chloroplasts (*uppercase letters*). Uppercase letters (*A* and *B*) correspond with those in Additional file 6: Figure S2 h. **a** Prior to embryogenesis. **b** Two-celled stage. **c** Early 4-celled stage. Daughter protoplasts do not rotate during cell divisions (**a**–**c**). **d** Mid 4-celled stage. **e** Late 4-celled stage. One pair of diagonally opposed daughter protoplasts (*A*) has shifted slightly toward the anterior of the embryo relative to the other pair (*B*) (**c**–**e**)

The time-lapse analysis from the anterior-lateral view of embryogenesis in *T. socialis* provided detailed insight into the behavior of daughter protoplasts (Fig. 3, Additional file 5). The daughter protoplasts did not rotate during successive cell divisions (Fig. 3a, b and c), which resulted in a 4-celled embryo with the longitudinal axes of the daughter protoplasts almost parallel to the anterior-posterior axis of the embryo (or the mother cell before embryogenesis; Fig. 3c). After successive cell divisions, one pair of diagonally opposed daughter protoplasts (corresponding to A and A’ in Additional file 6: Figure S2 h) shifted slightly toward the anterior of the embryo relative to the other pair (corresponding to B and B′ in Additional file 6: Figure S2 h; Fig. 3c, d and e). During this process, each daughter protoplast gradually emitted flagella from its apical end and changed its shape from a quarter of an ellipsoid to ovoid, without forming acute chloroplast ends (Fig. 3c, d and e).

### Indirect immunofluorescence microscopy of basal bodies, nuclei, and microtubules

**Fig. 4:**
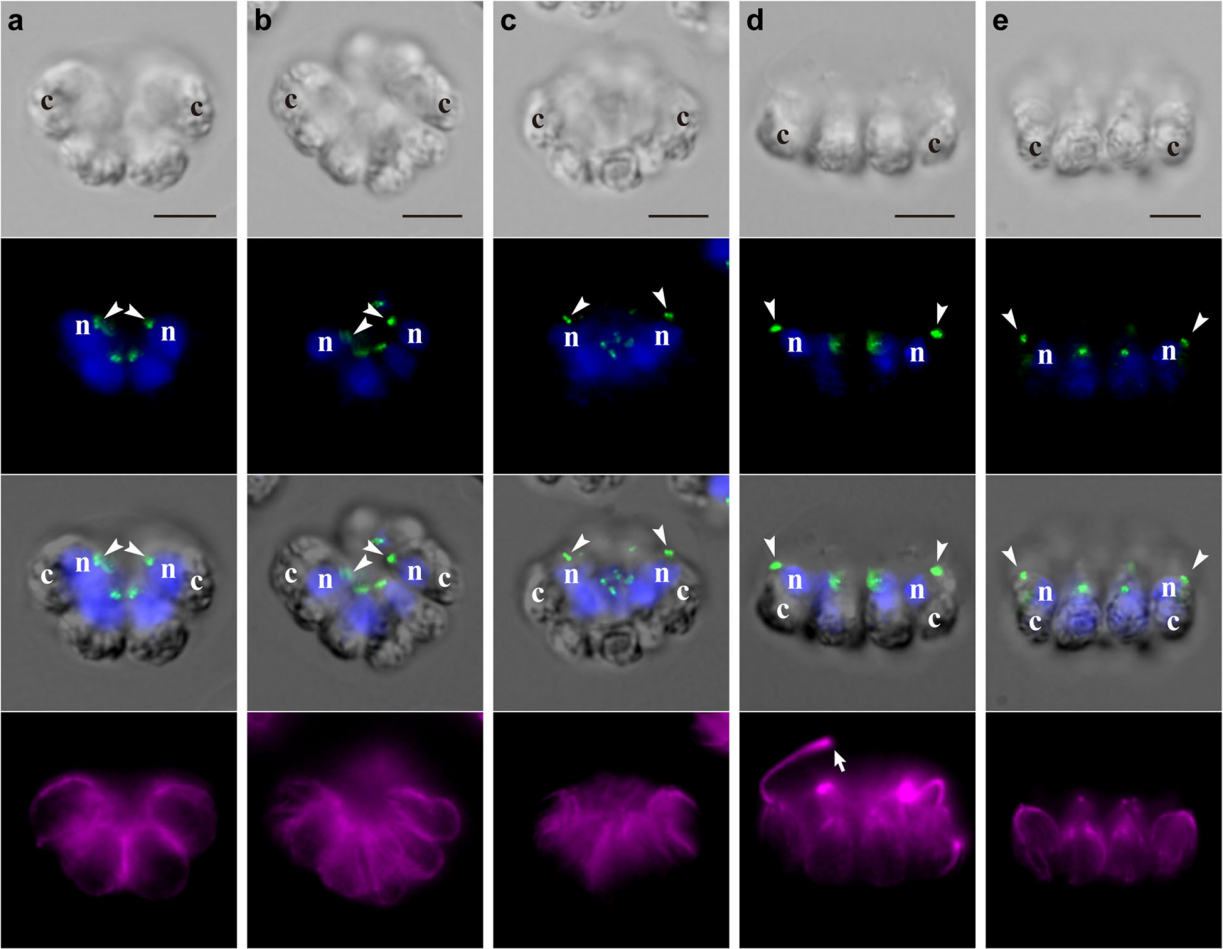
Indirect immunofluorescence microscopy showing successive stages of embryogenesis in *G. pectorale*. Each column shows a differential interference contrast (DIC) image (*top row*), a fluorescence image labeled with anti-SAS-6 antibody (green) and DAPI (blue) (*second row*), a merged DIC and fluorescence image of anti-SAS-6 antibody and DAPI (*third row*), and a fluorescence image labeled with anti-tubulin α antibody (magenta) of the same embryo. Positions of nuclei (*letter n*), chloroplasts (*letter c*), and basal bodies labeled with the anti-SAS-6 antibody (*arrowheads*) are shown. Scale bars: 5 μm. **a** Early 8-celled stage. **b** Late 8-celled stage. **c** Early 16-celled stage, just after the successive cell divisions. Basal bodies of daughter protoplasts are positioned in the center of the concave surface of the cell layer during successive cell divisions (**a**–**c**). **d** Mid 16-celled stage during partial inversion showing emitted flagella (*arrow*). The basal bodies of peripheral daughter protoplasts (*arrowheads*) have become separated from each other and are located slightly outside of the position of the nuclei. **e** After hatching. The basal bodies of peripheral cells (*arrowheads*) point toward the outside of the daughter colony

**Fig. 5:**
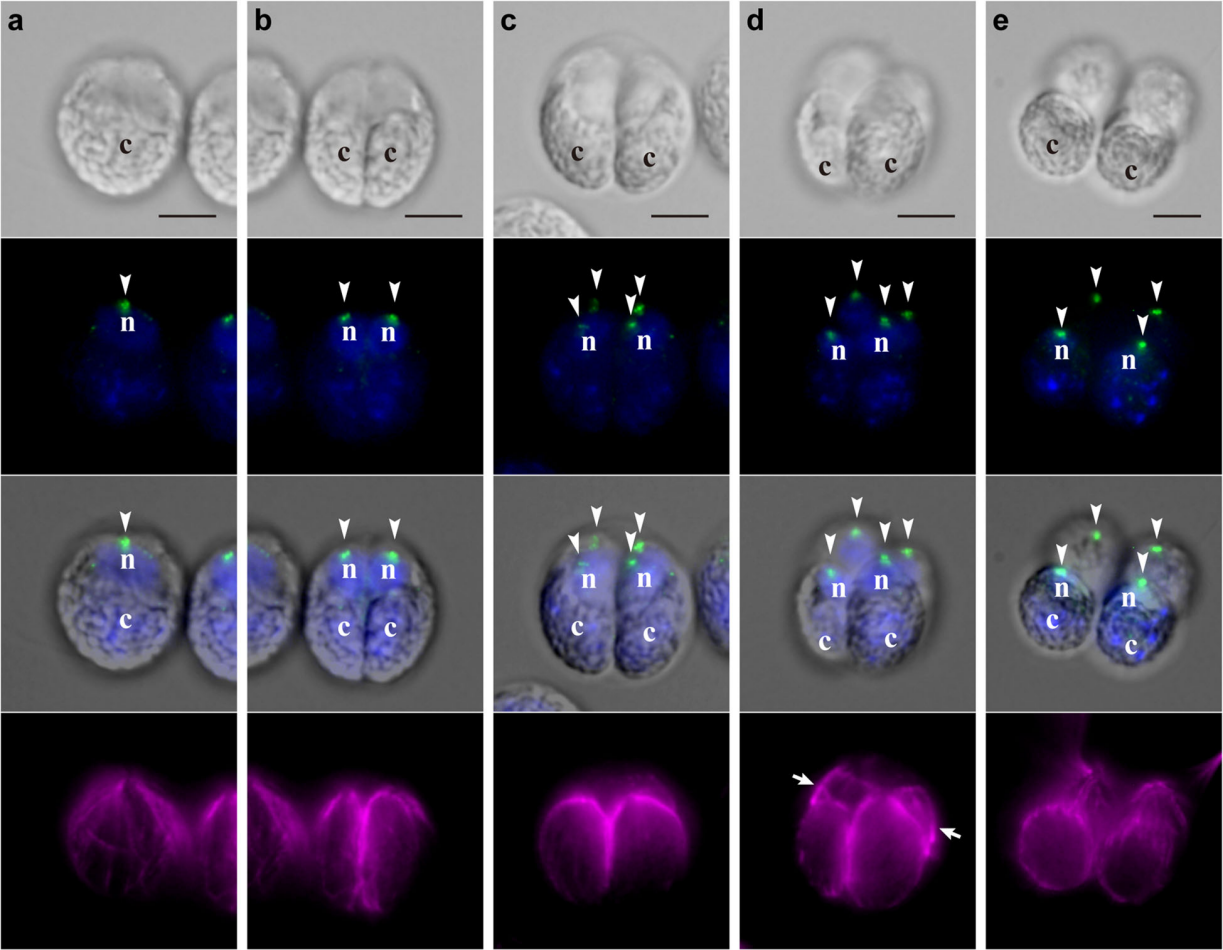
Indirect immunofluorescence microscopy showing successive stages of embryogenesis in *T. socialis*. Each column shows a DIC image (*top row*), a fluorescence image labeled with anti-SAS-6 antibody (green) and DAPI (blue) (*second row*), a merged DIC and fluorescence image of anti-SAS-6 antibody and DAPI (*third row*), and a fluorescence image labeled with anti-tubulin α antibody (magenta) of the same embryo. Positions of nuclei (*letter n*), chloroplasts (*letter c*), and basal bodies labeled with anti-SAS-6 antibody (*arrowheads*) are shown. Scale bars: 5 μm. **a** Prior to embryogenesis. **b** Two-celled stage. **c** Early 4-celled stage. The angles of the longitudinal axes of daughter protoplasts, which are indicated by basal bodies and chloroplasts, did not change during successive cell divisions (**a**–**c**). **d** Late 4-celled stage showing emitted flagella (*arrows*). The angles of longitudinal axes of daughter protoplasts did not change after successive cell divisions, though a pair of diagonally opposed daughter protoplasts shifted slightly toward the anterior of the embryo (“A” in Fig. 3c, e). **e** After hatching. The basal bodies of four cells are arranged in a square shape in the same plane

Cellular behaviors during embryogenesis in *G. pectorale* and *T. socialis* were examined at the subcellular level using indirect immunofluorescence microscopy of basal bodies, nuclei, and microtubules. The two basal bodies and two pro-basal bodies in each cell were indicated as four dots by localization of SAS-6 (see Methods). The locations of basal bodies and nuclei (stained with DAPI), as well as chloroplasts (observed in DIC images), clearly showed the longitudinal axis of each daughter protoplast during embryogenesis (Figs. 4 and 5). For identification of the stages of embryogenesis, an antibody against tubulin α was also used to stain cortical microtubules and the flagella that developed after successive cell divisions.

The staining of basal bodies allowed visualization of the expansion of the apical surface of the concave or cup-shaped 16-celled embryo in *G. pectorale* (Fig. 4). During successive cell divisions, the basal bodies of daughter protoplasts were located within the central region of the cup-shaped embryos and did not change their positions much (Fig. 4a, b and c). This situation of the basal bodies during successive divisions is consistent with the absence of rotation of daughter protoplasts observed in time-lapse imaging (Fig. 2a, b and c). After successive cell divisions, the basal bodies of the 12 peripheral protoplasts positioned in the center of the cup-shaped embryo moved from the center to the periphery in accordance with expansion of the cell layer of embryos (Fig. 4d). The basal bodies of each protoplast were positioned more peripherally than the longitudinal axis of each daughter protoplast (Fig. 4d, e). After daughter colonies hatched from the mother cell wall, the basal bodies of their peripheral cells were further separated from one another and pointed outward (Fig. 4e), but they did not move toward the posterior region as in successive cell divisions of *Astrephomene* [7].

In contrast to the apparent movement of the basal bodies in *G. pectorale*, the basal bodies of daughter protoplasts did not show obvious movement during embryogenesis in *T. socialis* (Fig. 5). During successive cell divisions, the basal bodies of daughter protoplasts were positioned in the anterior face of the embryo (Fig. 5a, b and c). After successive cell divisions, the basal bodies of a pair of diagonally opposed daughter protoplasts (corresponding to A and A’ in Additional file 6: Figure S2 h) shifted slightly toward the anterior relative to those in the other pair (corresponding to B and B′ in Additional file 6: Figure S2 h; Fig. 5d). No significant movement of the basal bodies was observed in developing embryos within the cell wall, except for a slight shifting parallel to the anterior-posterior axis of the embryo. After daughter colonies hatched out from the mother cell wall, we observed no shifting of their cells, and the basal bodies of four cells were arranged to form a square shape in the same plane (Fig. 5e), which suggests that flattening and extension of the cell layer occurred after hatching.

The morphological changes in the cell layer after successive cell divisions during embryogenesis in *G. pectorale* and *T. socialis* described above were verified quantitatively by measuring the distance between basal bodies relative to the diameter of the embryo/daughter colony (Additional file 6: Figures S3 and S4). In *G. pectorale*, the ratio of the distance between basal bodies of a diagonally opposed pair of peripheral protoplasts to the diameter of the 16-celled embryo/daughter colony increased significantly after the formation of flagella (Additional file 6: Figure S3a, b). It further increased after the daughter colony hatched from the mother cell wall (Additional file 6: Figure S3c, d). In *T. socialis*, on the other hand, the ratio of the distance between the basal bodies of two pairs of diagonally opposed protoplasts/cells to the diameter of the 4-celled embryo/daughter colony did not increase following the formation of flagella before hatching (Additional file 6: Figure S4a, b, d, e), but increased after hatching in both pairs of diagonally opposed cells (Additional file 6: Figure S4c, f, g).

## Discussion

**Fig. 6:**
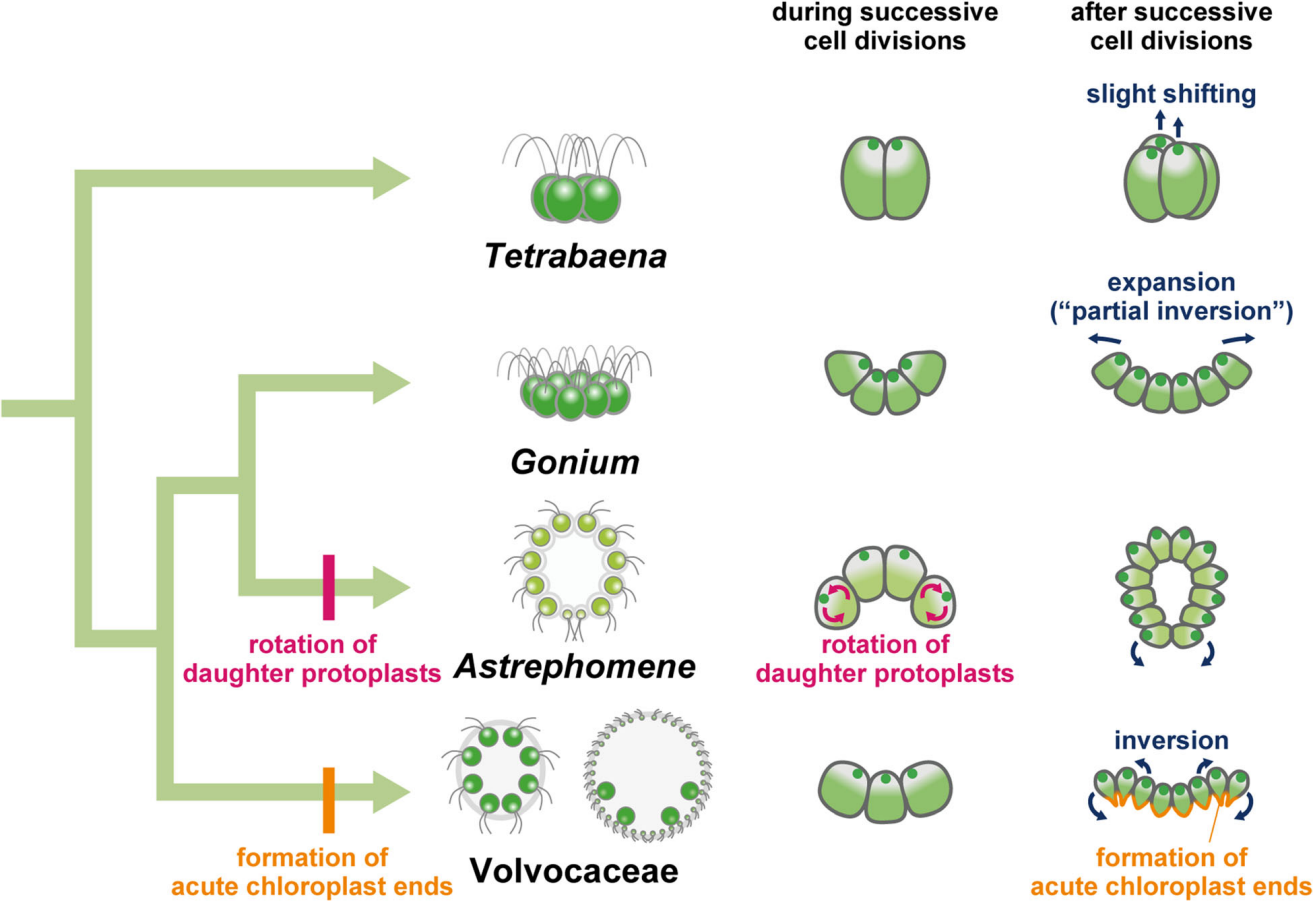
Schematic diagrams of the most likely evolutionary pathways of embryogenesis in volvocine green algae. Diagrams of stages of embryogenesis in *Astrephomene* and Volvocaceae (*Eudorina*) shown are based on a previous study [7], while those in *Tetrabaena* and *Gonium* are based on the present study (summarized in Additional file 6: Figure S5). Volvocacean species undergo the formation of acute chloroplast ends of daughter protoplasts, which is one of the principal factors producing the force for folding the cell layer during inversion after successive cell divisions. On the other hand, *Astrephomene* undergoes rotation of daughter protoplasts during successive cell divisions to form a spheroidal shape of the cell layer. Neither *Gonium* nor *Tetrabaena* showed the formation of acute chloroplast ends of daughter protoplasts after successive cell divisions or the rotation of daughter protoplasts during successive cell divisions. These results suggest that the ancestor of *Astrephomene* developed the rotation of daughter protoplasts after it diverged from the ancestors of *Gonium*, while the ancestor of Volvocaceae acquired the formation of acute chloroplast ends after it diverged from the ancestors of Goniaceae

Our results clearly showed a fundamental difference in the cellular mechanisms of embryogenesis between ancestral flattened colonies, *G. pectorale* and *T. socialis* (Additional file 6: Figure S5), and spheroidal colonies in the volvocine lineage (Additional file 6: Figure S1). Neither *G. pectorale* nor *T. socialis* showed the rotation of daughter protoplasts observed during successive cell divisions in *Astrephomene* (Figs. 2a, b and c, 3a, b and c, 4a, b and c and 5a, b and c, Additional files 2 and 5) [7]. Moreover, though the daughter protoplasts of *G. pectorale* and *T. socialis* underwent slight changes in shape, they did not display the formation of acute chloroplast ends after cell division that is seen during inversion in volvocacean species (Figs. 2c, d and e, 3c, d and e, 4c, d and e and 5c, d and e, Additional files 2 and 5) [7, 20, 23–31]. Considering these developmental characteristics along with the phylogenetic relationships of these volvocine lineages (Fig. 1) [5], the most likely scenario for the evolution of these cellular mechanisms in association with the emergence of spheroidal colonies is that the ancestor of *Astrephomene* developed the rotation of daughter protoplasts after it diverged from the ancestors of *Gonium*, while the ancestor of Volvocaceae acquired the formation of acute chloroplast ends after it diverged from the ancestors of Goniaceae (Fig. 6).

What were the molecular or genetic changes leading to the developmental modifications underlying the evolution of these body plans? One of the molecular bases associated with the formation of acute chloroplast ends in Volvocaceae is the regulation of cortical microtubules. In flask-shaped cells during inversion in *V*. *carteri*, the elongated stalks at the chloroplast ends are lined with cortical microtubules [20]. Moreover, colchicine (which prevents the assembly of microtubules) and cold treatment (which causes rapid disassembly of microtubules) have both been reported to block the formation of stalks, which suggests that the microtubules play an important role in the formation of stalks in flask-shaped cells [21]. Similar patterns of microtubules lining the acute chloroplast ends during inversion have also been observed in *V. tertius* [23] and *Eudorina elegans* [28]*.* By contrast, the indirect immunofluorescence microscopy used to stain the microtubules of embryos in *G. pectorale* and *T. socialis* in the present study did not demonstrate the distinctive pattern of cortical microtubules at the chloroplast ends; the cortical microtubules lined the entire cell bodies and concentrated around the apical ends after successive cell divisions (Figs. 4c, d and 5c, d). Such a difference in the patterning of cortical microtubules between volvocacean species and the two species with flattened colonies, *G. pectorale* and *T. socialis*, suggests that the ancestral genes involved in the remodeling or the maintenance of cortical microtubules, which have not previously been reported, have been co-opted or new genes have been acquired to form the acute chloroplast ends in the ancestor of the Volvocaceae.

Moreover, our results clearly demonstrate the details of the formation of flattened colonies in *G. pectorale* and *T. socialis* (Additional file 6: Figure S5). A recent study on embryogenesis in *G. pectorale* reported that a “partial inversion” occurred within the mother cell wall [33], while earlier studies reported that the flattening of the cell layer coincided with the breaking of the mother cell wall or hatching of the daughter colony [35, 38]. Our time-lapse analyses are consistent with both: the cup-shaped embryos formed after successive cell divisions expanded gradually within the mother cell wall (Fig. 2c, d and e, Additional file 2) and the further flattening of the cell layer occurred simultaneously with hatching (Additional file 3). Immunofluorescence microscopy staining the basal bodies during these stages also allowed clear visualization of these developmental processes (Fig. 4c, d and e; Additional file 6: Figure S3). Such a stepwise formation of flattened colonies was also observed in the embryogenesis of *T. socialis*: the slight shifting between the two pairs of diagonally opposed protoplasts occurred within the mother cell wall (Figs. 3c, d and e and 5c, d; Additional file 5) and the cell layer was flattened after hatching (Fig. 5e; Additional file 6: Figure S4).

These developmental processes in *G. pectorale* and *T. socialis* might be accompanied by the molecular bases which have been co-opted for the spheroidal colony formation in Volvocaceae and *Astrephomene*. During inversion in *V*. *carteri*, InvA, a type of kinesin localized in cytoplasmic bridges, interacts with cortical microtubules and moves cell bodies relative to cytoplasmic bridges, which results in the production of the driving force for folding the cell sheet [39]. Previous studies using transmission electron microscopy did not observe such a relocation of cytoplasmic bridges in *G. pectorale* and *T. socialis* [33, 34]*.* However, the genome data of both species include putative homologs of *InvA* (GPECTOR_36g129, Acc. No. KXZ47277.1 in the *G. pectorale* genome [15] and Kinesin-like protein KIF27, partial, Acc. No. PNH03015.1 in the *T. socialis* genome [16], only a partial sequence), and these putative homologs might be involved in development, such as in expansion of the cell layer in *G. pectorale* or the shifting of protoplasts in *T. socialis*. Another two known genes in *V*. *carteri*, *InvB* and *InvC*, encode a GDP-mannose transporter and a glycosyltransferase, respectively, which are involved in enlargement of the gonidial vesicle of the extracellular matrix that provides sufficient space for inversion [40, 41]. The enlargement of the mother cell wall after successive cell divisions was observed in *G. pectorale* (Fig. 2c–e), and several genes encoding proteins similar to InvB and InvC also exist in the genome of *G. pectorale* [15] and *T. socialis* [16]*.* Thus, the genes involved in the enlargement of the mother cell wall in the volvocacean ancestor with flattened colonies, like those in *Gonium* spp., might have been co-opted to support inversion in the extant volvocacean species. These hypotheses regarding the co-option of ancestral genes for the molecular mechanisms of spheroidal colony formation may be verified by further investigation using recently established techniques such as transformation of *G. pectorale* [42] and its application to *T. socialis*.

## Conclusion

Using light microscopy time-lapse imaging and staining cellular components with indirect immunofluorescence microscopy, we described the formation of flattened colonies in *G. pectorale* and *T. socialis* in detail at cellular and subcellular levels. Compared to species with spheroidal colonies, *G. pectorale* and *T. socialis* showed simpler embryogenesis, without the rotation of daughter protoplasts during successive cell divisions and the formation of acute chloroplast ends after successive cell divisions, implying that these mechanisms were independently acquired by the distinct ancestors of species that form spheroidal colonies. Although some molecular mechanisms of inversion have been investigated in *Volvox*, the molecular or genetic bases underlying the rotation of daughter protoplasts in *Astrephomene* are currently unknown. Further analyses of these cellular mechanisms in Volvocaceae and *Astrephomene* and comparative analyses with *Gonium* and *Tetrabaena* would unveil the molecular and genetic bases, and the modification of these, which brought about the evolution of the body plan from flattened to spheroidal colonies.

## Methods

### Strains and culture conditions

We used *T. socialis* strain NIES-571 [43] to observe embryogenesis in *Tetrabaena*. As the *Gonium* strains used in recent genome [15] and morphological studies [34, 44] frequently show abnormal morphology in vegetative colonies and no formation of completely flattened 16-celled colonies (data not shown), we established several new strains of *Gonium*. We used one of these new strains, *G. pectorale* strain 2017–0423-IsgGo1, in the present study (Additional file 6: Figure S6). The culture was grown in 10 mL of SVM medium [45] in screw-capped tubes (18 × 150 mm) and maintained at 25 °C on a 12-h light/12-h dark schedule under cool-white fluorescent lamps at an intensity of 50–90 μmol·m^− 2^·s^− 1^. Species identification was based on morphological and molecular data (Additional file 6: Figures S6 and S7).

For developmental observation, *G. pectorale* strain 2017–0423-IsgGo1 was grown synchronously in silicon-capped 500-mL Erlenmeyer flasks containing 250 mL SVM medium with aeration at 32 °C on a 16-h light/8-h dark schedule under cool-white fluorescent lamps at an intensity of 140–180 μmol·m^− 2^·s^− 1^, and *T. socialis* strain NIES-571 was grown synchronously in silicon-capped 500 mL Erlenmeyer flasks containing 250 mL SVM medium with aeration at 20 °C on a 12-h light/12-h dark schedule under cool-white fluorescent lamps at an intensity of 240–280 μmol·m^− 2^·s^− 1^.

### Light microscopy time-lapse imaging

Embryogenesis of *G. pectorale* strain 2017–0423-IsgGo1 and *T. socialis* strain NIES-571 was observed by time-lapse light microscopy based on methods reported previously [7, 34] with some modifications. As the polyethyleneimine coating on coverslips hampered the normal development of *G. pectorale* and *T. socialis* embryos (data not shown), we used coverslips without coating. Culture containing fully mature vegetative colonies of *G. pectorale* or *T. socialis* was placed on slides, and then the coverslips, surrounded by thin banks of petroleum jelly, were placed on top of the culture. In this method, cells and embryos are attached to the coverslip with weak adhesion of the flagella or cell walls. Preparations were observed under a BX-53 microscope (Olympus, Tokyo, Japan) equipped with Nomarski interference optics. Photomicrographs were obtained using DP Controller 1.2.1108 (Olympus) at 1-min (*G. pectorale*) or 2-min (*T. socialis*) intervals. This difference in timing was implemented in response to the observation that *T. socialis* embryos ceased embryogenesis when photomicrographs were taken at 1-min intervals, likely because they are sensitive to light and/or heat, so that frequent exposure to light under light microscopy prevented *T. socialis* embryos from proceeding with their development. As the embryos of *G. pectorale* and *T. socialis* moved and rotated during observation, positions and angles of images were adjusted manually using Adobe Photoshop CC (Adobe Systems Inc., San Jose, CA, USA). The sequence of adjusted images was analyzed and processed using ImageJ 1.50b (National Institutes of Health, Bethesda, MD, USA). Embryogenesis was observed at least three times in each species and showed almost identical patterns in each observation.

### Indirect immunofluorescence microscopy

To verify the behavior of daughter protoplasts during embryogenesis in *G. pectorale* and *T. socialis*, immunostaining of basal bodies and microtubules was initially performed as previously described [34]. However, we found that the cortical microtubules of *G. pectorale* and *T. socialis* were fragile and broke when subjected to that protocol, and thus we introduced the following modifications. The samples were placed in fixing solution-A (3.7% formaldehyde [Sigma-Aldrich, St. Louis, MO, USA], 0.1% Triton X-100 [Sigma-Aldrich], and 1 mM DTT [Nacalai Tesque Inc., Kyoto, Japan] in phosphate-buffered saline [PBS]) for 1 min and then into fixing solution-B (3.7% formaldehyde and 1 mM DTT in PBS; i.e., fixing solution-A without Triton X-100) for 14 min, instead of the original protocol of 15 min in fixing solution-A. Chlorophyll and other pigments in the samples were extracted in − 20 °C methanol for 1 min, instead of extracting solution [34] for 60 min. The samples were then subjected to two primary antibodies and two secondary antibodies, each for 1 h at 37 °C. The primary antibodies were a rat anti-tubulin α antibody (clone YL1/2, Bio-Rad Laboratories, Inc., Hercules, CA, USA) and a rabbit anti-CrSAS-6 antibody [46] diluted 1:500 with blocking buffer (0.44% gelatin [Sigma-Aldrich], 0.05% NaN_3_, and 1% bovine serum albumin [Sigma-Aldrich] in PBS). The secondary antibodies were an Alexa Fluor 568-conjugated goat anti-rat IgG (Invitrogen) and an Alexa Fluor 488-conjugated goat anti-rabbit IgG (Invitrogen) diluted 1:500 with the blocking buffer described above. The samples were placed on a drop of SlowFade Gold Antifade Mountant (Thermo Fisher Scientific Inc., Waltham, MA, USA) mixed with 0.5 μL of 0.1 mg/mL DAPI (Sigma-Aldrich). Preparations were observed under a BX-60 microscope (Olympus) equipped with Nomarski interference optics, a mercury lamp, and filter sets with DP Controller 1.2.1108 (Olympus). Differential interference contrast and fluorescence images were merged using Adobe Photoshop CC (Adobe Systems Inc.). For quantitative analysis of the shapes of embryos in successive developmental stages, we measured the diameter of the embryo/daughter colony in both species, as well as the distance between basal bodies of a diagonally opposed pair of peripheral protoplasts/cells in *G. pectorale* or a diagonally opposed pair of protoplasts/cells in *T. socialis*, using ImageJ 1.50b (National Institutes of Health).

## Additional files

Additional file 6: **Figure S1.** Schematic diagrams of embryogenesis in Volvocaceae and *Astrephomene*. **Figure S2.** Cleavage patterns and cell lineages in embryogenesis of *G. pectorale* and *T. socialis*. **Figure S3.** Measurement of morphological changes of the cell layer in 16-celled embryos/daughter colonies during colonial development in *G. pectorale*. **Figure S4.** Measurement of morphological changes of the cell layer in 4-celled embryos/daughter colonies during development in *T. socialis*. **Figure S5.** Schematic diagrams of embryogenesis in *Gonium* and *Tetrabaena*. **Figure S6.** Vegetative colonies in *G. pectorale* strain 2017-0423-IsgGo1. **Figure S7.** Bayesian phylogenetic tree of *Gonium* based on *rbcL* genes. **Table S1.** List of *rbcL* gene used in the phylogenetic analysis of *Gonium*. (PDF 6884 kb)

## Data Availability

The new *rbcL* sequence of *G*. *pectorale* strain 2017–0423-IsgGo1 has been deposited to DDBJ/EMBL/GenBank (accession number: LC459976). The sequence alignment used for molecular identification of the new strain (Additional file 6: Figure S7 and Table S1) has been deposited in TreeBASE (https://treebase.org/treebase-web/home.html; study ID: 23894). *G*. *pectorale* strain 2017–0423-IsgGo1 are available from Microbial Culture Collection at the Institute for National Environmental Studies (http://mcc.nies.go.jp/localeAction.do?lang=en) as NIES-4327. All the other data generated or analyzed in this study are included in this article and its Additional files.

## Abbreviations

DAPI: 4′,6-diamidino-2-phenylindole
DIC: Differential interference contrast
DTT: Dithiothreitol
PBS: Phosphate-buffered saline

## References

[1] Michod RE (2006). On the transfer of fitness from the cell to the multicellular organism. Biol Philos.

[2] Grosberg RK, Strathmann RR (2007). The evolution of multicellularity: a minor major transition?. Annu Rev Ecol Evol Syst.

[3] Rensing SA (2016). (why) does evolution favour embryogenesis?. Trends Plant Sci.

[4] Nozaki H, Ito M (1994). Phylogenetic relationships within the colonial Volvocales (Chlorophyta) inferred from cladistic analysis based on morphological data. J Phycol.

[5] Nozaki H, Misawa K, Kajita T, Kato M, Nohara S, Watanabe MM (2000). Origin and evolution of the colonial Volvocales (Chlorophyceae) as inferred from multiple, chloroplast gene sequences. Mol Phylogenet Evol.

[6] Herron MD, Michod RE (2008). Evolution of complexity in the volvocine algae: transitions in individuality through Darwin’s eye. Evolution.

[7] Yamashita S, Arakaki Y, Kawai-Toyooka H, Noga A, Hirono M, Nozaki H (2016). Alternative evolution of a spheroidal colony in volvocine algae: developmental analysis of embryogenesis in *Astrephomene* (Volvocales, Chlorophyta). BMC Evol Biol.

[8] Kirk DL (2005). A twelve-step program for evolving multicellularity and a division of labor. BioEssays.

[9] Herron MD, Hackett JD, Aylward FO, Michod RE (2009). Triassic origin and early radiation of multicellular volvocine algae. Proc Natl Acad Sci U S A.

[10] dos Reis M, Thawornwattana Y, Angelis K, Telford MJ, Donoghue PCJ, Yang Z (2015). Uncertainty in the timing of origin of animals and the limits of precision in molecular timescales. Curr Biol.

[11] Dohrmann M, Wörheide G (2017). Dating early animal evolution using phylogenomic data. Sci Rep.

[12] Sánchez-Baracaldo P, Raven JA, Pisani D, Knoll AH (2017). Early photosynthetic eukaryotes inhabited low-salinity habitats. Proc Natl Acad Sci.

[13] Merchant SS, Prochnik SE, Vallon O, Harris EH, Karpowicz SJ, Witman GB (2007). The *Chlamydomonas* genome reveals the evolution of key animal and plant functions. Science.

[14] Prochnik SE, Umen J, Nedelcu AM, Hallmann A, Miller SM, Nishii I (2010). Genomic analysis of organismal complexity in the multicellular green alga *Volvox carteri*. Science.

[15] Hanschen ER, Marriage TN, Ferris PJ, Hamaji T, Toyoda A, Fujiyama A (2016). The *Gonium pectorale* genome demonstrates co-option of cell cycle regulation during the evolution of multicellularity. Nat Commun.

[16] Featherston J, Arakaki Y, Hanschen ER, Ferris PJ, Michod RE, Olson BJSC (2018). The 4-celled *Tetrabaena socialis* nuclear genome reveals the essential components for genetic control of cell number at the origin of multicellularity in the volvocine lineage. Mol Biol Evol.

[17] Hamaji T, Kawai-Toyooka H, Uchimura H, Suzuki M, Noguchi H, Minakuchi Y (2018). Anisogamy evolved with a reduced sex-determining region in volvocine green algae. Commun Biol.

[18] Sugasawa M, Matsuzaki R, Arakaki Y, Nozaki H (2015). Morphology and phylogenetic position of a rare four-celled green alga, *Pascherina tetras* (Volvocales, Chlorophyceae), based on cultured material. Phycologia.

[19] Munakata H, Nakada T, Nakahigashi K, Nozaki H, Tomita M (2016). Phylogenetic position and molecular chronology of a colonial green flagellate, *Stephanosphaera pluvialis* (Volvocales, Chlorophyceae), among unicellular algae. J Eukaryot Mirobiol.

[20] Viamontes GI, Kirk DL (1977). Cell shape changes and the mechanism of inversion in *Volvox*. J Cell Biol.

[21] Viamontes GI, Fochtmann LJ, Kirk DL (1979). Morphogenesis in *Volvox*: analysis of critical variables. Cell.

[22] Green K, Viamontes GI, Kirk DL (1981). Mechanism of formation, ultrastructure, and function of the cytoplasmic bridge system during morphogenesis in *Volvox*. J Cell Biol.

[23] Pickett-Heaps JD (1970). Some ultrastructural features of *Volvox*, with particular reference to the phenomenon of inversion. Planta.

[24] Kelland JL (1977). Inversion in *Volvox* (Chlorophyceae). J Phycol.

[25] Ireland GW, Hawkins SE (1981). Inversion in *Volvox tertius*: the effects of con a. J Cell Sci.

[26] Höhn S, Hallmann A (2011). There is more than one way to turn a spherical cellular monolayer inside out: type B embryo inversion in *Volvox globator*. BMC Biol.

[27] Gottlieb B, Goldstein ME (1977). Colony development in *Eudorina elegans* (Chlorophyta, Volvocales). J Phycol.

[28] Marchant HJ (1977). Colony formation and inversion in the green alga *Eudorina elegans*. Protoplasma.

[29] Fulton AB (1978). Colonial development in *Pandorina morum*: II Colony morphogenesis and formation of the extracellular matrix. Dev Biol.

[30] Iida H, Nishii I, Inouye I (2011). Embryogenesis and cell positioning in *Platydorina caudata* (Volvocaceae, Chlorophyta). Phycologia.

[31] Höhn S, Hallmann A (2016). Distinct shape-shifting regimes of bowl-shaped cell sheets – embryonic inversion in the multicellular green alga *Pleodorina*. BMC Dev Biol.

[32] Hallmann A (2006). Morphogenesis in the family Volvocaceae: different tactics for turning an embryo right-side out. Protist.

[33] Iida H, Ota S, Inouye I (2013). Cleavage, incomplete inversion, and cytoplasmic bridges in *Gonium pectorale* (Volvocales, Chlorophyta). J Plant Res.

[34] Arakaki Y, Kawai-Toyooka H, Hamamura Y, Higashiyama T, Noga A, Hirono M (2013). The simplest integrated multicellular organism unveiled. PLoS One.

[35] Harper RA (1912). The structure and development of the colony in *Gonium*. Trans Am Microsc Soc.

[36] Gerisch G (1959). Die Zelldifferenzierung bei *Pleodorina californica* Shaw und die Organisation der Phytomonadinenkolonien. Arch Für Protistenkd.

[37] Hayama M, Nakada T, Hamaji T, Nozaki H (2010). Morphology, molecular phylogeny and taxonomy of *Gonium maiaprilis* sp. nov. (Goniaceae, Chlorophyta) from Japan. Phycologia.

[38] Hartmann M (1924). Über die Veränderung der Koloniebildung von *Eudorina elegans* und *Gonium pectorale* unter dem Einfluß äußerer Bedingungen. IV. Mitt. der Untersuchungen über die Morphologie und Physiologie des Formwechsels der Phytomonadinen (Volvocales). Arch Für Protistenkd.

[39] Nishii I, Ogihara S, Kirk DL (2003). A kinesin, InvA, plays an essential role in *Volvox* morphogenesis. Cell.

[40] Ueki N, Nishii I (2008). *Idaten* is a new cold-inducible transposon of *Volvox carteri* that can be used for tagging developmentally important genes. Genetics.

[41] Ueki N, Nishii I (2009). Controlled enlargement of the glycoprotein vesicle surrounding a *Volvox* embryo requires the InvB nucleotide-sugar transporter and is required for normal morphogenesis. Plant Cell.

[42] Lerche K, Hallmann A (2009). Stable nuclear transformation of *Gonium pectorale*. BMC Biotechnol.

[43] Kasai F, Kawachi M, Erata M, Mori F, Yumoto K, Sato M (2009). NIES-collection list of strains, 8th edition. Jpn J Phycol Sôrui.

[44] Arakaki Y, Fujiwara T, Kawai-Toyooka H, Kawafune K, Featherston J, Durand PM (2017). Evolution of cytokinesis-related protein localization during the emergence of multicellularity in volvocine green algae. BMC Evol Biol.

[45] Kirk DL, Kirk MM (1983). Protein synthetic patterns during the asexual life cycle of *Volvox carteri*. Dev Biol.

[46] Nakazawa Y, Hiraki M, Kamiya R, Hirono M (2007). SAS-6 is a cartwheel protein that establishes the 9-fold symmetry of the centriole. Curr Biol.

