# Supplementary material for: Embryogenesis of flattened colonies implies the innovation required for the evolution of spheroidal colonies in volvocine green algae
Source: BMC Evol Biol. 2019 Jun 11;19:120. doi: 10.1186/s12862-019-1452-x (PMC6560780; doi:10.1186/s12862-019-1452-x)
Supplement: Supplementary file 6 — Figure S1. Schematic diagrams of embryogenesis in Volvocaceae and Astrephomene. Figure S2. Cleavage patterns and cell lineages in embryogenesis of G. pectorale and T. socialis. Figure S3. Measurement of morphological changes of the cell layer in 16-celled embryos/daughter colonies during colonial development in G. pectorale. Figure S4. Measurement of morphological changes of the cell layer in 4-celled embryos/daughter colonies during development in T. socialis. Figure S5. Schematic diagrams of embryogenesis in Gonium and Tetrabaena. Figure S6. Vegetative colonies in G. pectorale strain 2017-0423-IsgGo1. Figure S7. Bayesian phylogenetic tree of Gonium based on rbcL genes. Table S1. List of rbcL gene used in the phylogenetic analysis of Gonium. (PDF 6884 kb) [file 12862_2019_1452_MOESM6_ESM.pdf]

Additional file 6: Figs. S1–S7 and Table S1

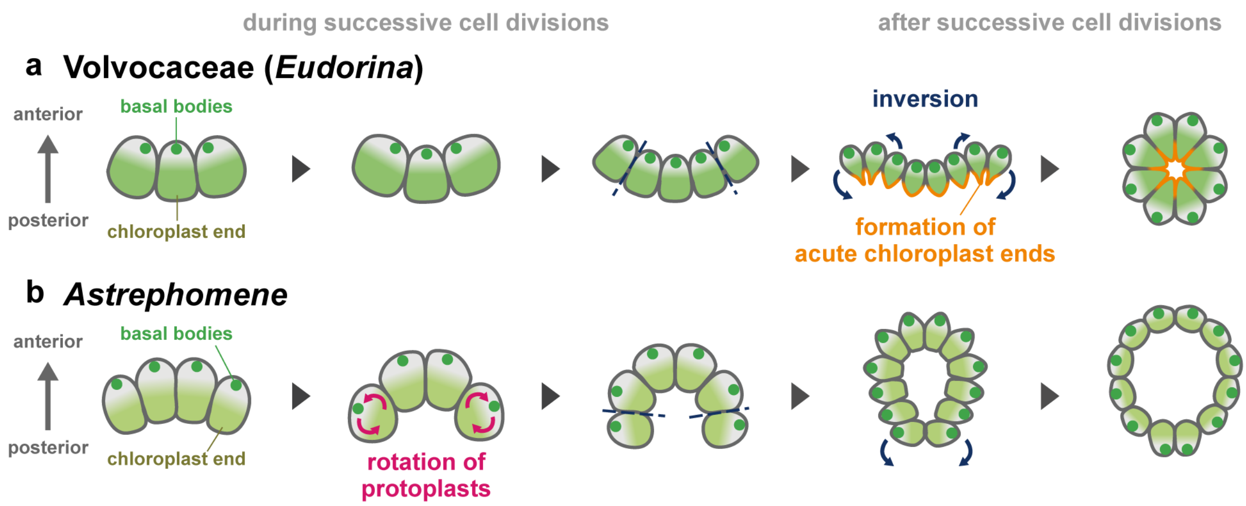

Fig. S1. Schematic diagrams of embryogenesis in Volvocaceae and *Astrephomene*. Diagrams are based on a previous study [1] and represent lateral views of stages of embryogenesis with anterior sides of embryos oriented toward the top of the figure. (**a**) Embryogenesis in *Eudorina* as a representative of Volvocaceae. A mature reproductive cell undergoes successive cell divisions and forms a cup-shaped embryo composed of a single layer of daughter protoplasts. Following the successive divisions, the embryo inverts its cell layer to form a spheroidal daughter colony, with the formation of acute chloroplast ends (opposite to basal bodies) and the relocation of cytoplasmic bridges at the chloroplast ends. (**b**) Embryogenesis in *Astrephomene*. In contrast to Volvocaceae, an embryo of *Astrephomene* undergoes rotation of daughter protoplasts during successive cell divisions and forms a nearly spheroidal cell layer just after successive cell divisions.

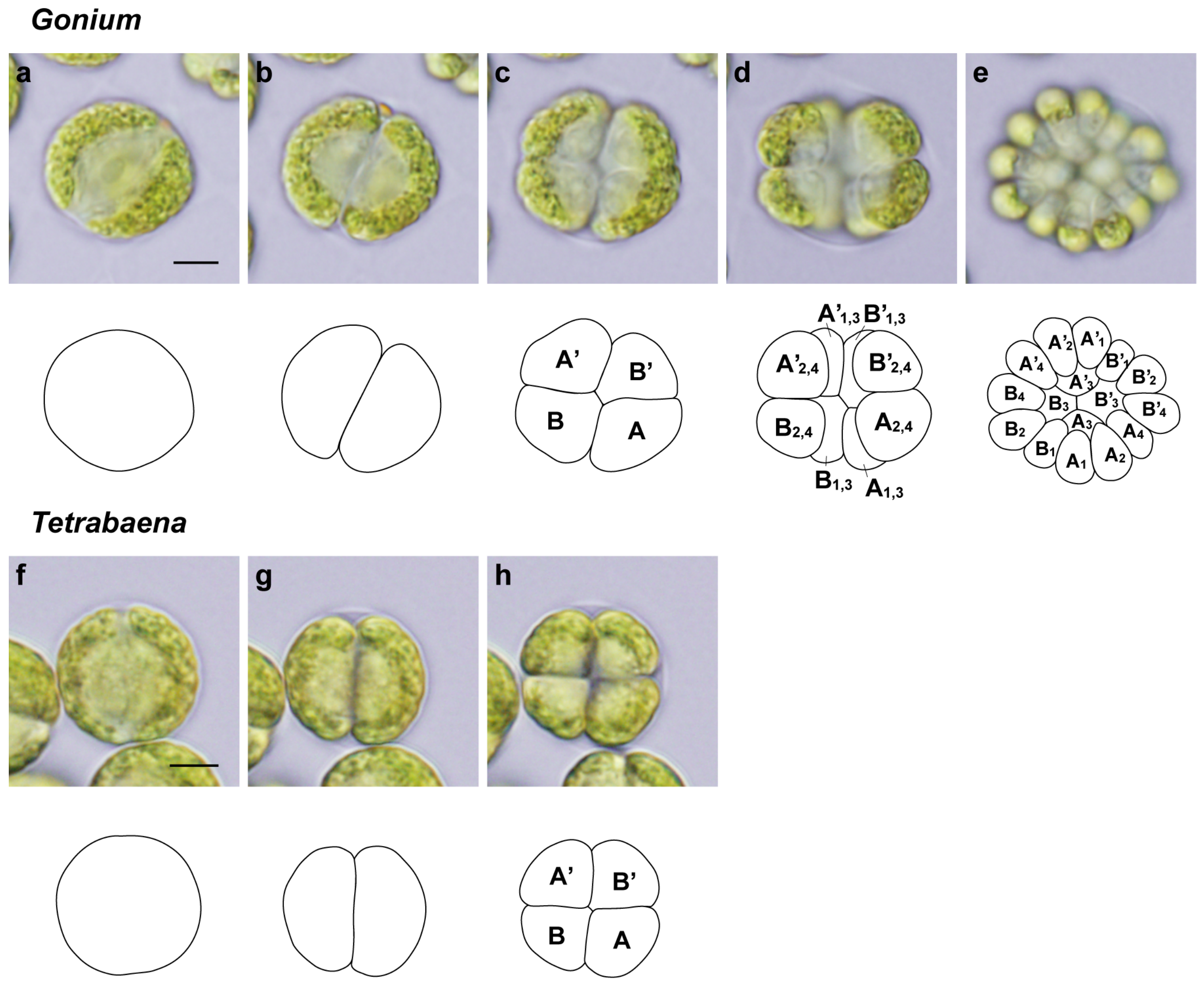

Fig. S2. Cleavage patterns and cell lineages in embryogenesis of *G. pectorale* and *T. socialis*. Based on the time-lapse imaging of each species (Additional files 1 and 4). Outlines of daughter protoplasts were traced from each image. All images in each row are at the same magnification. Scale bars: 5 µm. (**a**–**e**) Successive images of embryogenesis in *G. pectorale* from the anterior view. (**a**) Prior to embryogenesis. (**b**) Two-celled stage. (**c**) 4-celled stage. (**d**) 8-celled stage. (**e**) 16-celled stage. Cleavage patterns were 180° rotationally symmetrical about the longitudinal axis of the embryo and essentially the same as described in previous studies [2–4]. The naming of each daughter protoplast followed that in *A. gubernaculifera* [1], since the cleavage patterns up to 16-celled stage of *G. pectorale* and *A. gubernaculifera* are almost identical except for the direction of daughter protoplasts [1,5]. (**f**–**h**) Successive images of embryogenesis in *T. socialis* from the anterior view. (**f**) Prior to embryogenesis. (**g**) Two-celled stage. (**h**) 4-celled stage. The cleavage patterns were essentially the same as described in a previous study [6]. Note that one pair of diagonally opposed daughter protoplasts were separated from each other (A and A’ in **h**) while the other pair were slightly attached to each other at the center of the embryo (B and B’ in **h**).

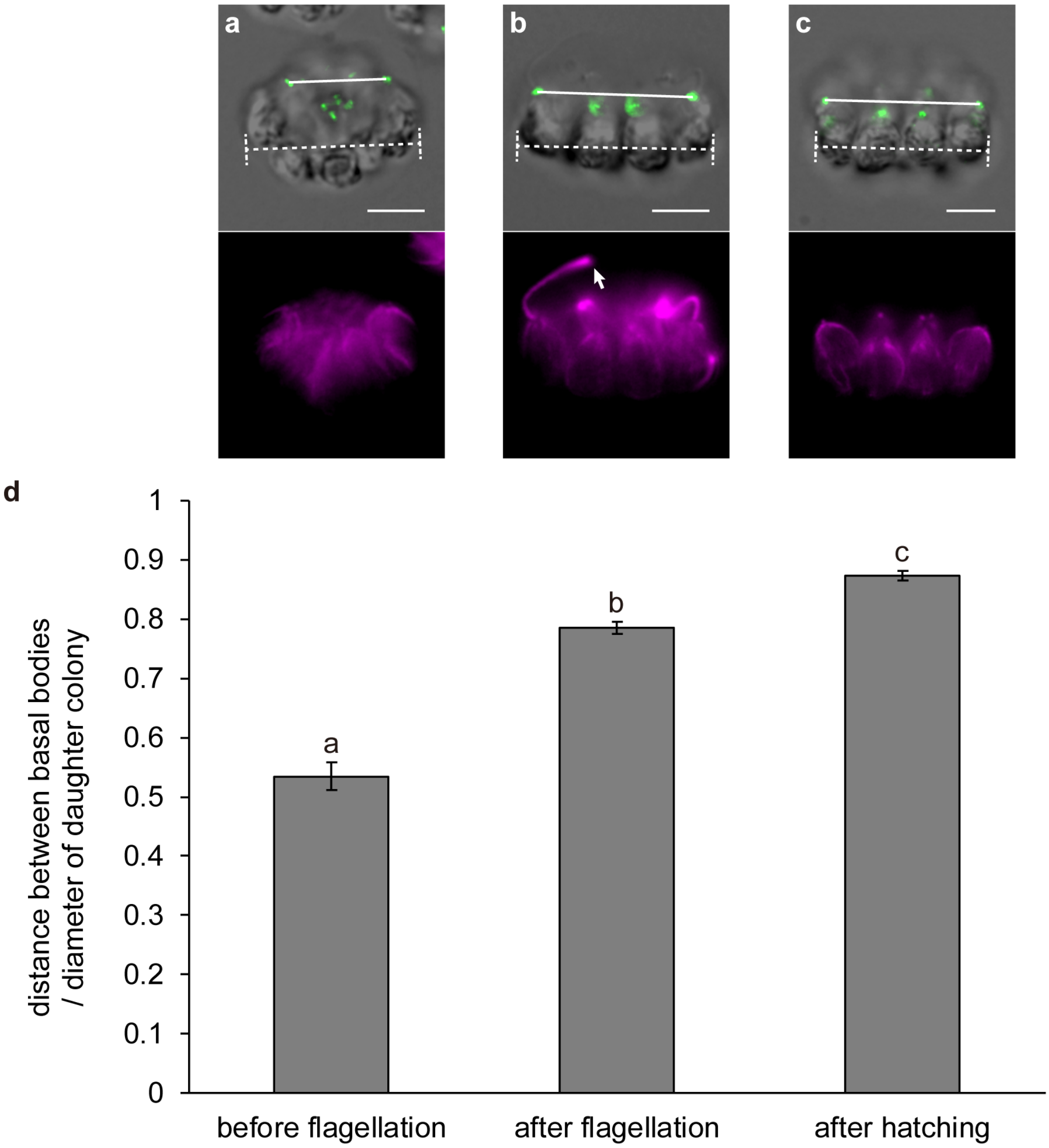

Fig. S3. Measurement of morphological changes of the cell layer in 16-celled embryos/daughter colonies during colonial development in *G. pectorale*. (**a**–**c**) Merged fluorescence images labeled with anti-SAS-6 antibody (green) and DIC images (*top row*), and fluorescence images labeled with anti-tubulin α antibody (magenta, *second row*) of examples of 16-celled embryos/daughter colonies before flagellation (**a**), after flagellation (**b**; *arrow* indicates flagellum), and after hatching (**c**). Scale bars: 5 μm. The distance between basal bodies of diagonally opposed pairs of peripheral protoplasts/cells (*solid lines*, without distinction of the location of the pair; see Fig. S2e) and the diameter of 16-celled embryos/daughter colonies (*dotted lines*) were measured at each of these stages. (**d**) The ratio of the distance between the basal bodies of a diagonally opposed pair of peripheral protoplasts/cells to the diameter of embryos/daughter colonies in three stages during development. The stages of development were distinguished as follows: before flagellation (with no flagella or flagella < 1 μm, n = 22), after flagellation (with flagella > 5 μm and a mother cell wall, n = 42), and after hatching (without a mother cell wall, n = 20). Bars represent means ± standard error (SE). Different letters above bars indicate significant differences of means based on a Tukey–Kramer post-hoc test at $P < 0.05$.

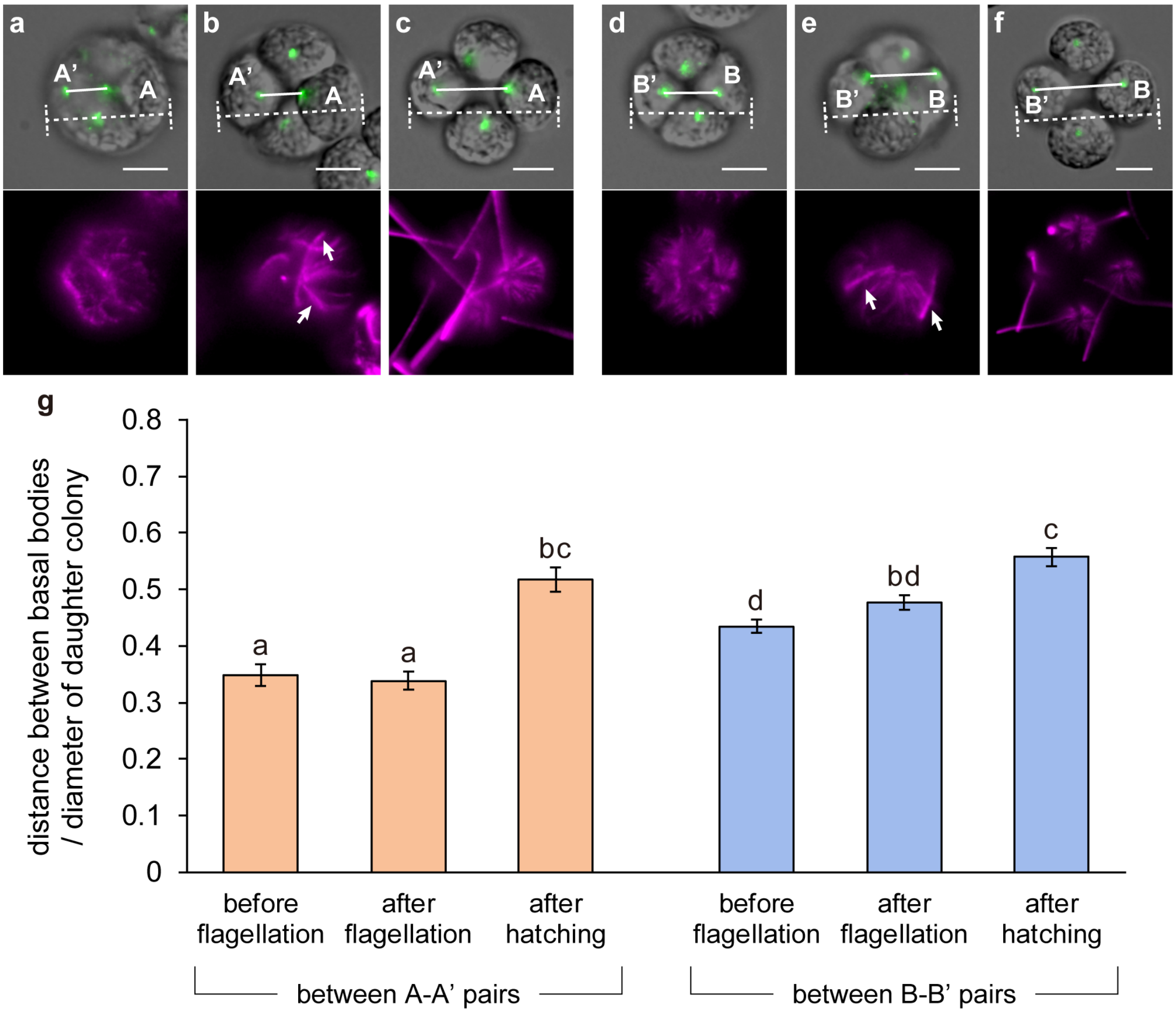

Fig. S4. Measurement of morphological changes of the cell layer in 4-celled embryos/daughter colonies during development in *T. socialis*. (**a**–**f**) Merged fluorescence images labeled with anti-SAS-6 antibody (green) and DIC images (*top row*), and fluorescence images labeled with anti-tubulin α antibody (magenta, *second row*) of examples of 4-celled embryos/daughter colonies before flagellation (**a**, **d**), after flagellation (**b**, **e**, *arrows* indicate flagella), and after hatching (**c**, **f**). Scale bars: 5 μm. The distance between basal bodies of a pair of diagonally opposed protoplasts/cells (*solid lines*) between A-A' pairs (**a**–**c**) or B-B' pairs (**d**–**f**; see Fig. S2h) and the diameter of embryos/daughter colonies (*dotted lines*) were measured at each of these stages. Uppercase letters (*A* and *B*) correspond with those in Fig. S2h. (**g**) The ratio of the distance between basal bodies of a pair of diagonally opposed protoplasts/cells to the diameter of 4-celled embryos/daughter colonies in three developmental stages, with distinction between the two pairs. The stages of development were distinguished as follows: before flagellation (with no flagella or flagella < 1 μm), after flagellation (with flagella > 5 μm and a mother cell wall), and after hatching (without a mother cell wall). Bars represent means ± SE (n = 15–21). As the distance of both pairs could not be measured in some daughter colonies, the distances between basal bodies of A-A' and B-B' cells were treated as independent data. Different letters above bars indicate significant differences of means based on a Tukey–Kramer post-hoc test at $P < 0.05$.

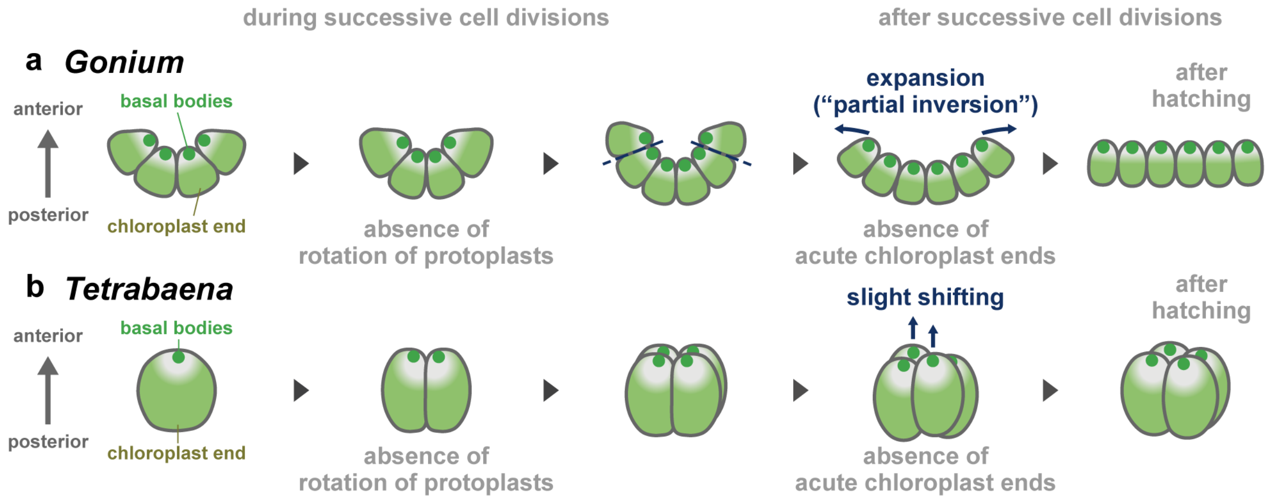

Fig. S5. Schematic diagrams of embryogenesis in *Gonium* and *Tetrabaena*. Diagrams are based on the present time-lapse analysis (Figs. 2 and 3) and indirect immunofluorescence microscopy (Figs. 4 and 5) and represent lateral views of stages of embryogenesis with anterior sides of embryos oriented toward the top of the figure. Note that the rotation of daughter protoplasts seen in *Astrephomene* and the formation of acute chloroplast ends of daughter protoplasts seen during inversion in the Volvocaceae were not observed during embryogenesis in *G. pectorale* and *T. socialis*. (**a**) Embryogenesis in *G. pectorale*. Each cell of a vegetative colony performed four successive cell divisions to form a cup-shaped 16-celled embryo with basal bodies positioned in the center of the concave surface of the cell layer. The cell layer of the embryo expanded gradually after successive cell divisions and instantly upon hatching of the daughter colony, with the basal bodies moving from the center to the periphery of the cell layer. (**b**) Embryogenesis in *T. socialis*. Each cell of a vegetative colony performed two successive cell divisions to form a 4-celled embryo with the basal bodies positioned in the anterior face of the embryo. The two pairs of diagonally opposed daughter protoplasts shifted slightly after successive cell divisions. The cell layer flattened and expanded after the daughter colony hatched.

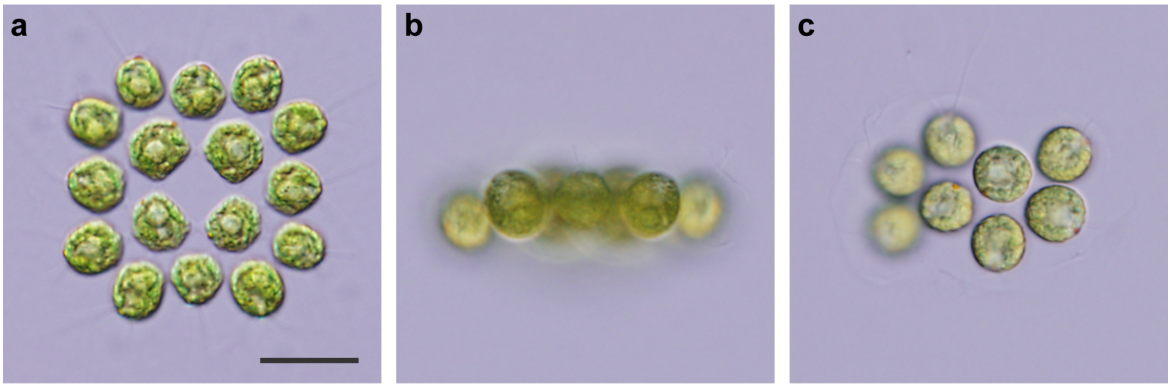

Fig. S6 Vegetative colonies in *G. pectorale* strain 2017-0423-IsgGo1. *G. pectorale* strain 2017-0423-IsgGo1 was isolated from a soil sample collected from a rice paddy field (24°24'54.9” N, 123°46'45.4” E) in Yaeyama, Okinawa Prefecture, Japan on May 20, 2017. The dried soil was rewetted with distilled water in Petri dishes (90 × 20 mm) and incubated at 25°C on a 12-h light/12-h dark schedule under cool-white fluorescent lamps at an intensity of 50–90 $\mu mol \cdot m^{-2} \cdot s^{-1}$. After 7–8 days, vegetative colonies appeared in the Petri dishes. Clones were established using the pipette-washing method [7]. For the morphological identification of species, light microscopy of mature vegetative colonies in two-days-old culture was carried out with a BX53 microscope (Olympus, Tokyo, Japan) equipped with Nomarski interference optics. All images are at the same magnification. Scale bar: 20 µm. (**a**) 16-celled colony from the anterior view. Each cell has only one pyrenoid. (**b**) 16-celled colony from the lateral view. (**c**) 8-celled colony from the anterior view. All 8-celled colonies in this strain consist of four zigzag rows of two cells each, which is a characteristic of *G. pectorale* [8].

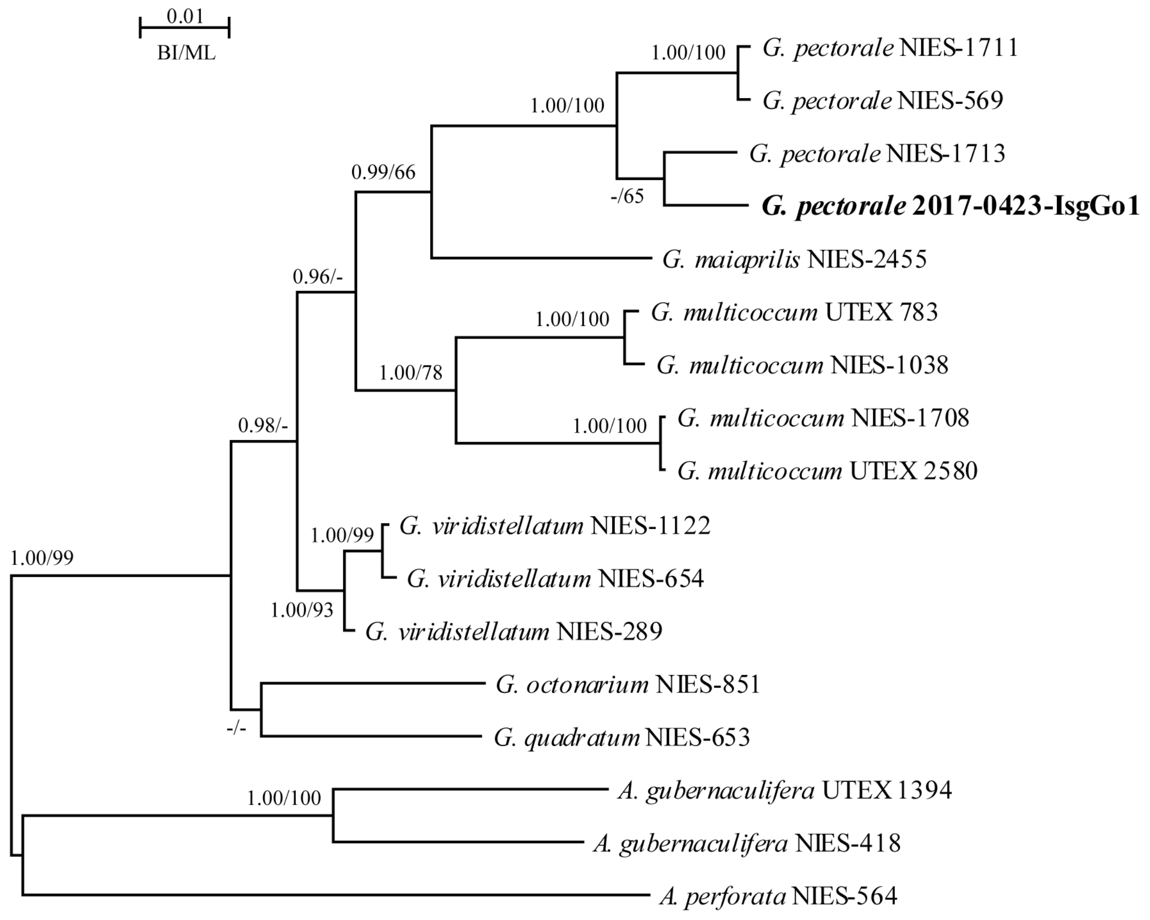

Fig. S7 Bayesian phylogenetic tree of *Gonium* based on *rbcL* genes. The coding region of *rbcL*, the chloroplast gene for the large subunit of RuBisCO, of *G. pectorale* strain 2017-0423-IsgGo1 were sequenced with essentially the same method in previous research [9–12]. The 1,128 bp corresponded to position 31-1,158 of *rbcL* from *G. pectorale* strain 2017-0423-IsgGo1 and 14 OTUs of *Gonium* as well as 3 OTUs of *Astrephomene* (as outgroup) (Table S1) were subjected to Bayesian interference and maximum-likelihood analysis. For the substitution model, GTR + I + G model were selected using MEGA 7.0.21 [13]. Bayesian interference was performed using MrBayes 3.2.6 [14] with 1,000,000 generations of Markov chain Monte Carlo iterations; the first 25% of the generations were discarded as burn-in. Maximum-likelihood analysis was performed with 1,000 replicates of bootstrap analyses [15] performed by MEGA 7.0.21 [13]. The posterior probabilities (≥ 0.95) from Bayesian interference (left) and bootstrap value (≥ 50%) from maximum-likelihood (right) are shown at branches.

Table S1: List of *rbcL* gene used in the phylogenetic analysis of *Gonium*.

Taxon	Strain designation	Accession number
Gonium pectorale	2017-0423-IsgGo1	LC459976[c]
	NIES[a]-569	D63437
	NIES-1713	AB246189
	NIES-1711	AB246190
Gonium maiaprilis	NIES-2455	AB520743
Gonium multicoccum	UTEX[b] 2580	D63435
	UTEX 783	AB076103
	NIES-1038	AB246187
	NIES-1708	AB246188
Gonium octonarium	NIES-851	D63436
Gonium quadratum	NIES-653	D63438
Gonium viridistellatum	NIES-654	D86831
	NIES-289	AB076091
	NIES-1122	AB076093
Astrephomene gubernaculifera	NIES-418	D63428
	UTEX 1394	AB044170
Astrephomene perforata	NIES-564	D63429

[a] Microbial Culture Collection at the National Institute for Environmental Studies [16].
[b] Culture Collection of Algae at the University of Texas at Austin [17].
[c] Sequenced in this study.

References for Additional file 6

1. Yamashita S, Arakaki Y, Kawai-Toyooka H, Noga A, Hirono M, Nozaki H. Alternative evolution of a spheroidal colony in volvocine algae: developmental analysis of embryogenesis in *Astrephomene* (Volvocales, Chlorophyta). BMC Evol Biol. 2016;16:243.
2. Harper RA. The structure and development of the colony in *Gonium*. Trans Am Microsc Soc. 1912;31:65–82.
3. Gerisch G. Die Zelldifferenzierung bei *Pleodorina californica* Shaw und die Organisation der Phytomonadinenkolonien. Arch Für Protistenkd. 1959;104:292–358.
4. Iida H, Ota S, Inouye I. Cleavage, incomplete inversion, and cytoplasmic bridges in *Gonium pectorale* (Volvocales, Chlorophyta). J Plant Res. 2013;126(5):699–707.
5. Nozaki H, Ito M. Phylogenetic relationships within the colonial Volvocales (Chlorophyta) inferred from cladistic analysis based on morphological data. J Phycol. 1994;30(2):353–65.
6. Arakaki Y, Kawai-Toyooka H, Hamamura Y, Higashiyama T, Noga A, Hirono M, et al. The simplest integrated multicellular organism unveiled. PLOS ONE. 2013;8(12):e81641.
7. Pringsheim EG. Pure Cultures of Algae. Cambridge: Cambridge University Press; 1946.
8. Hayama M, Nakada T, Hamaji T, Nozaki H. Morphology, molecular phylogeny and taxonomy of *Gonium maiaprilis* sp. nov. (Goniaceae, Chlorophyta) from Japan. Phycologia. 2010;49(3):221–34.
9. Fawley MW, Fawley KP. A simple and rapid technique for the isolation of DNA from microalgae. J Phycol. 2004;40(1):223–5.
10. Nakada T, Nozaki H. Re-evaluation of three *Chlorogonium* (Volvocales, Chlorophyceae) species based on 18S ribosomal RNA gene phylogeny. Eur J Phycol. 2007;42(2):177–82.
11. Nozaki H, Ito M, Sano R, Uchida H, Watanabe MM, Kuroiwa T. Phylogenetic relationships within the colonial Volvocales (Chlorophyta) inferred from *rbc*L gene sequence data. J Phycol. 1995;31(6):970–9.
12. Nozaki H, Ito M, Sano R, Uchida H, Watanabe MM, Takahashi H, et al. Phylogenetic analysis of *Yamagishiella* and *Platydorina* (Volvocaceae, Chlorophyta) based on *rbc*L gene sequences. J Phycol. 1997;33(2):272–8.
13. Kumar S, Stecher G, Tamura K. MEGA7: Molecular Evolutionary Genetics Analysis Version 7.0 for Bigger Datasets. Mol Biol Evol. 2016;33(7):1870–4.
14. Ronquist F, Teslenko M, van der Mark P, Ayres DL, Darling A, Hohna S, et al. MrBayes 3.2: Efficient Bayesian Phylogenetic Inference and Model Choice Across a Large Model Space. Syst Biol. 2012;61(3):539–42.
15. Felsenstein J. Confidence limits on phylogenies: an approach using the bootstrap. Evolution. 1985;39(4):783–91.
16. Kasai F, Kawachi M, Erata M, Mori F, Yumoto K, Sato M, et al. NIES-collection list of strains, 8th edition. Jpn J Phycol Sôrui. 2009;57(1, Supplement):1–350.
17. Starr RC, Zeikus JA. UTEX—The culture collection of algae at the University of Texas at Austin 1993 List of Cultures. J Phycol. 1993;29(s2):1–106.
